# Evaluating the Inheritance Risk: Epilepsy Prevalence among Offspring of Adults with Epilepsy in a Tertiary Referral Epilepsy Center

**DOI:** 10.3390/jcm13102932

**Published:** 2024-05-16

**Authors:** Tassanai Intravooth, Hazal Baran, Anne-Sophie Wendling, Amjad Halaby, Bernhard J. Steinhoff

**Affiliations:** 1 Kork Epilepsy Center, Landstr. 1, 77694 Kehl-Kork, Germany; tintravooth@epilepsiezentrum.de (T.I.); hbaran@epilepsiezentrum.de (H.B.); awendling@epilepsiezentrum.de (A.-S.W.); amjad.halaby91@gmail.com (A.H.); 2 Medical Faculty, University of Freiburg, Breisacher Str. 64, 79106 Freiburg, Germany

**Keywords:** epilepsy, offspring, prevalence, risk, outpatient clinic, idiopathic generalized epilepsy

## Abstract

While significant strides have been made in comprehending the pathophysiology and treatment of epilepsy, further investigation is warranted to elucidate the factors impacting its development and transmission, particularly within familial contexts. This study sought to explore the prevalence and risk factors associated with epilepsy in the offspring of patients with epilepsy who were treated at a tertiary epilepsy center. Adult patients with confirmed epilepsy (PWE) receiving outpatient care were consecutively enrolled, starting from January 2021 to January 2023. Data were recorded for various variables, including age, gender, epilepsy pathophysiology, cognitive impairment, and family history of epilepsy. Descriptive statistics, various statistical tests, and multivariate logistic regression analyses were employed to analyze the data. A total of 1456 PWE were included. Among them, 463 patients (31.8%) had children. Twenty-five patients had offspring diagnosed with epilepsy, representing a prevalence of 5.4%. Analysis of the offspring with epilepsy revealed older ages, a higher proportion of parents with idiopathic epilepsy, and a greater prevalence of a positive family history of epilepsy. Multivariate logistic regression analysis demonstrated a significant association between a family history of epilepsy and increased epilepsy risk in offspring. Genetic syndrome-immanent predisposition, advanced age, and a family history of epilepsy were identified as significant risk factors for epilepsy in offspring by means of this mono-center study.

## 1. Introduction

Epilepsy, a neurological disorder characterized by recurrent seizures, affects millions of individuals worldwide, imposing significant physical, psychological, and socioeconomic burdens [1]. While considerable progress has been made in understanding the pathophysiology and management of epilepsy, there remains a need for further exploration into the factors influencing its development and transmission, particularly in familial contexts.

The risk of epilepsy in offspring of patients with epilepsy (PWE) has long been recognized, suggesting a potential genetic predisposition [2,3,4]. Moreover, the presence of a positive family history emerges as a potent predictor of epilepsy risk, spanning both idiopathic epilepsy and specific subtypes of focal epilepsies [5,6,7,8,9,10,11,12,13,14]. However, the precise mechanisms underlying epilepsy inheritance and transmission remain incompletely understood.

Additionally, demographic and clinical factors may also contribute to epilepsy risk in offspring. Parental age, for instance, has been implicated as a potential contributing factor, with advanced parental age possibly correlating with an elevated risk of epilepsy in their children [15]. Similarly, parental gender may influence the transmission of epilepsy, although the specifics of this relationship warrant further exploration [2,3]. Moreover, the presence of comorbidities in parents, such as certain neurological or psychiatric disorders, could potentially heighten the risk of epilepsy in their offspring, underscoring the multifaceted nature of epilepsy etiology [16,17,18].

The study presented here marks the initial phase of a broader investigation. Following this initial phase, we aim to monitor the offspring of our PWE based on the antiseizure medication their mothers took during pregnancy. We plan to assess the psychiatric and neurocognitive of these offspring through neuropsychological and psychiatric evaluations. However, further funding and approval from the local ethical committee are required for these subsequent studies.

This study aims to investigate the prevalence and risk factors associated with epilepsy in offspring of PWE, utilizing a comprehensive approach encompassing descriptive analysis and multivariate logistic regression. By elucidating the interplay of genetic, demographic, and clinical factors, we seek to enhance our understanding of epilepsy inheritance and inform strategies for risk assessment, prevention, and intervention.

## 2. Material and Methods

### 2.1. Study Design and Participants

This was a cross sectional study using data between January 2021 and January 2023 at the Kork Epilepsy Center, Germany. The Kork Epilepsy Center is one of the main tertiary referral epilepsy centers in Germany.

We recruited eligible out-patients with a definite diagnosis of epilepsy, as defined by the International League Against Epilepsy (ILAE) criteria [1]. These were PWE with focal, generalized, or unclassified epilepsy who visited our out-patients department. Seizure-free patients and those with other comorbidities were also included in the study. However, patients presenting solely with psychogenic non-epileptic seizures or other seizure-like symptoms in the outpatient clinic without a confirmed diagnosis of epilepsy were excluded. Upon returning their signed informed consent forms, the recruited PWE and their offspring were treated and interviewed by one of the authors (BJS).

### 2.2. Variables and Data Sources

Recorded data included the age of PWE at the time of their offspring’s birth, the gender of PWE, the survey date, epilepsy pathophysiology, epilepsy classification, presence of cognitive impairment, and whether patients had other family members with epilepsy, as well as whether they had children. For patients with children, information such as the number of offspring, occurrence of epilepsy in these children, and the age of the offspring at the time of the survey was documented. Data regarding cognitive impairment were obtained from medical records or surveys. However, the definition of cognitive impairment was not specified or standardized across records. Indeed, all recruited PWE were outpatients regularly treated by BJS. In addition to our outpatient investigations, which encompassed a comprehensive medical history, assessment of the seizure situation, routine EEG on the day of the appointment, and laboratory assessments, an additional approximately 30 min were dedicated specifically to the study for all PWE.

### 2.3. Statistical Analysis

The collected data were transferred to and analyzed using Excel version 16.65 (Microsoft 365). Individual statistical analyses were conducted using JASP (Jeffreys’s Amazing Statistics Program). Descriptive statistics, including means, standard deviations, and percentage distributions, were employed to summarize the data. To address missing data on offspring age, linear regression was utilized with the PWE’s age as a predictor variable (y = 1.0134x − 31.448, where y = children’s age and x = parents’ age). In cases where the calculated age was smaller than zero, such values were replaced with zero. Two offspring had passed away, so we replaced their ages with the ages at the time of their death.

Continuous variables in the basic characteristics were analyzed using Student’s *t*-test, while categorical variables were assessed using the Chi-square test or Fisher’s test and presented as frequency values (%). Multivariate logistic regression analyses were employed to analyze the potential risk factors for epilepsy. A significance level of *p* < 0.05 was considered statistically significant.

### 2.4. Ethics

The study was conducted in accordance with the principles outlined in the International Conference on Harmonisation (ICH) guidelines and the Declaration of Helsinki. Approval for the study was obtained from the ethics committee of the Albert-Ludwigs-University of Freiburg (EK-Freiburg 22-1425-S1, date of approval 05JAN2023), and it was registered on the German Clinical Trials Register website under ID no. DRKS00031459. Written informed consent for study participation was obtained from all participants, including PWE and their offspring.

## 3. Results

### 3.1. Descriptive Data of the PWE

A total of 1456 patients were included (age range: 18–89 years, mean age: 43 years, 47.6% male). Out of the total sample, 463 patients (31.8%), of whom 44% were male, had offspring (range: 1–4 offspring, median: 2). Among the 1456 patients, 289 were identified as having cognitive impairment. Among them, only four patients (1.4%) were found to have offspring. All offspring of patients with cognitive impairment (*n* = 10) were found to have no epilepsy. Among the patients diagnosed with idiopathic generalized epilepsy (*n* = 113), 13 (11.5%) had offspring diagnosed with epilepsy. In contrast, among parents with other types of epilepsy (focal or not clearly classifiable as focal or generalized) (*n* = 350), the percentage of offspring diagnosed with epilepsy was 3.4% (12/350). A total of 25 patients had offspring diagnosed with epilepsy, representing a prevalence of 5.4%. Among them, fourteen patients (ten women and four men) had children after being diagnosed with epilepsy. Six women took antiseizure medication during pregnancy. Additionally, six patients (three women and three men) had children before being diagnosed with epilepsy. For the remaining five patients, we do not have precise information.

### 3.2. Analysis of the Offspring

Among the 463 patients, a total of 823 offspring were reported. Among them, 29 were diagnosed with epilepsy (3.5%). Offspring with a diagnosis of epilepsy were older (30 vs. 25, *p* = 0.021) compared to those without epilepsy. Additionally, they had a higher proportion of parents classified as having idiopathic epilepsy (41% vs. 23%, *p* = 0.045) and a higher proportion of other family members diagnosed with epilepsy (93% vs. 8%, *p* < 0.001). Both groups of offspring did not differ significantly in terms of parental gender, parental cognitive impairment, or parental age at the time of birth (see Table 1)

### 3.3. Multivariate Logistic Regression Analysis of Epilepsy Risk Factors in Offspring of PWE

All offspring of patients with cognitive impairment were found to have no epilepsy (*n* = 0); therefore, this variable could not be included in the logistic model. As shown in Table 2, after adjusting for other factors, the odds ratio (OR) for epilepsy among individuals with a family history of epilepsy was 240 times higher than among those without such a history (*p* < 0.001). Additionally, the risk of epilepsy in offspring increased by a factor of 0.04 for every year of age (*p* = 0.021). In this logistic model, there was no significant difference in the risk of epilepsy between the offspring of parents diagnosed with idiopathic epilepsy compared to those with other diagnoses (*p* = 0.184).

## 4. Discussion

The findings of this study shed light on various factors associated with epilepsy risk in offspring of PWE. Notably, the analysis of descriptive data revealed a higher prevalence of epilepsy among the offspring of parents diagnosed with idiopathic generalized epilepsy compared to those with other types of epilepsy. Furthermore, our study identified advanced age, a family history of epilepsy, and parental classification of idiopathic epilepsy as significant risk factors for epilepsy in offspring.

While there have been scarce reports regarding the epilepsy risk of offspring of parents with cognitive impairment, one might speculate about the potential presence of a genetic disorder that could lead to more severe cognitive disability and seizures in the offspring [18]. However, in our study, all offspring of patients with cognitive impairment were free of epilepsy. Although the small sample size limits conclusive interpretation, this intriguing finding warrants further investigation into the interplay between cognitive impairment and epilepsy risk in offspring.

It is noteworthy, though not surprising, that the percentage of PWE who experienced cognitive impairment and had offspring was remarkably low. This observation raises an interesting point about societal attitudes towards epilepsy and its associated challenges. It is a curious paradox that while there is often considerable concern about the potential cognitive effects of antiseizure medications on offspring, comparatively little attention is paid to the cognitive issues experienced by patients themselves.

Moreover, there is an intriguing aspect to consider regarding the reproductive choices of individuals with cognitive impairment. Research has shown that individuals with cognitive impairments generally have fewer children than those without such impairments [19,20,21,22]. This disparity in reproductive patterns could further contribute to the lower percentage of PWE with cognitive impairment who have offspring. Therefore, this observation prompts us to reflect on broader societal attitudes towards epilepsy and cognitive impairment. It highlights the need for greater awareness, support, and advocacy for individuals living with epilepsy, including those facing cognitive challenges. By addressing these issues holistically, we can work towards fostering a more inclusive and supportive environment for all individuals affected by epilepsy.

The multivariate logistic regression analysis revealed the significant impact of a positive family history on epilepsy risk in offspring, indicating an odds ratio 240 times higher in individuals with such a history. These findings are supported by several other studies in the literature. Numerous investigations have documented an elevated risk of epilepsy incidence among patients with a positive family history [5,7,11,12,14], particularly in cases of idiopathic epilepsy [23,24]. Additionally, certain types of focal epilepsies have been associated with a positive family history [6,8,9,10,13]. These findings underscore the role of genetic predisposition in epilepsy etiology and emphasize the importance of genetic counseling and screening in families with a history of epilepsy. In our subsequent study, we will further explore and monitor this association, examining its nuances and potential implications.

Moreover, our findings indicate a modest but significant increase in epilepsy risk with advancing age of offspring. This underscores the importance of longitudinal monitoring and early intervention strategies in at-risk populations to mitigate the burden of epilepsy.

Interestingly, we found no significant difference in epilepsy risk between offspring of parents diagnosed with idiopathic epilepsy and those with other types of epilepsy after adjusting for other factors. This suggests that while genetic factors do play a prominent role, other factors such as environmental influences and epigenetic mechanisms may also contribute to epilepsy risk in offspring.

While our study provides valuable insights into the prevalence and risk factors associated with epilepsy in offspring of patients with epilepsy, it is important to acknowledge several limitations. Firstly, the study was conducted at a single institution, which may limit the generalizability of the findings to other populations or settings. Additionally, the sample size of our study was relatively small, which may have impacted the statistical power to detect significant associations or trends. Furthermore, the retrospective nature of the study design may have introduced bias or confounding factors that were not accounted for in the analysis. Additionally, data on certain variables, such as cognitive impairment, were obtained from medical records or surveys, which may have introduced inaccuracies or incomplete information. Lastly, the study focused primarily on the association between epilepsy risk and familial factors, and other potential risk factors, such as environmental exposures or comorbidities, were not fully explored. Despite these limitations, our study provides important insights into the complex interplay of genetic and familial factors in epilepsy risk and underscores the need for further research in this area.

## 5. Conclusions

In conclusion, our study provides valuable insights into the complex interplay of genetic, demographic, and clinical factors influencing epilepsy risk in offspring of PWE. However, further research incorporating larger cohorts and longitudinal follow-up is warranted to validate these findings and elucidate the underlying mechanisms driving epilepsy inheritance and transmission.

## Figures and Tables

**Table 1:** Characteristics of offspring with epilepsy patients.

	Epilepsy (*n* = 29)	No Epilepsy (*n* = 794)	*p*
Parent’s gender			0.674
Male	15 (52%)	365 (46%)	
Female	14 (48%)	429 (54%)	
Age (years) Mean (SD)			
Offspring	30 (12)	25 (16)	**0.021**
Parent	31 (5)	31 (6)	0.819
Parent’s classification of epilepsy			**0.045**
Idiopathic	12 (41%)	186 (23%)	
Other	17 (59%)	608 (77%)	
Parental cognitive impairment			0.999 *
Yes	0 (0%)	10 (1%)	
No	29 (100%)	784 (99%)	
Family history of epilepsy			<0.001 *
Yes	27 (93%)	66 (8%)	
No	2 (7%)	728 (92%)	

* Fisher’s test.

**Table 2:** Multivariate logistic regression of epilepsy risk factors among offspring of PWE.

	Multivariate Logistic Regression OR (95% CI)	*p*
Parent’s gender		
Male	1.45 (0.53–3.97)	0.474
Female	Ref	
Age		
Offspring	1.04 (1.01–1.08)	**0.021**
Parent	0.99 (0.92–1.08)	0.948
Parent’s classification of epilepsy		
Idiopathic	0.53 (0.20–1.36)	0.184
Other	Ref	
Family history of epilepsy		
Yes	240 (51–1123)	**<0.001**
No	Ref	

## Data Availability

Data were pseudonymized for the statistical analysis. Raw data are stored at the archive of the Kork Epilepsy Center.
